# Exome Sequencing and Prediction of Long-Term Kidney Allograft Function

**DOI:** 10.1371/journal.pcbi.1005088

**Published:** 2016-09-29

**Authors:** Laurent Mesnard, Thangamani Muthukumar, Maren Burbach, Carol Li, Huimin Shang, Darshana Dadhania, John R. Lee, Vijay K. Sharma, Jenny Xiang, Caroline Suberbielle, Maryvonnick Carmagnat, Nacera Ouali, Eric Rondeau, John J. Friedewald, Michael M. Abecassis, Manikkam Suthanthiran, Fabien Campagne

**Affiliations:** 1 The HRH Prince Alwaleed Bin Talal Bin Abdulaziz Alsaud Institute for Computational Biomedicine, Weill Cornell Medical College, New York, New York, United States of America; Department of Physiology and Biophysics, The Weill Cornell Medical College, New York, New York, United States of America; 2 INSERM UMR1155 et Service des Urgences Néphrologiques et Transplantation Rénale, APHP, Hôpital Tenon, Paris, France; 3 Sorbonne Universités, UPMC Université Paris 06, Paris, France; 4 Division of Nephrology and Hypertension, Weill Cornell Medical College, New York, New York, United States of America; 5 Department of Transplantation Medicine, New York Presbyterian Hospital, New York, New York, United States of America; 6 Genomics Core Facility, Weill Cornell Medical College, New York, New York, United States of America; 7 Laboratoire d'histocompatibilité Hôpital Saint Louis APHP, Paris, France; 8 Northwestern University Feinberg School of Medicine, Chicago, Illinois, United States of America; 9 Comprehensive Transplant Center, Northwestern University Feinberg School of Medicine, Chicago, Illinois, United States of America; La Jolla Institute for Allergy and Immunology, UNITED STATES

## Abstract

Current strategies to improve graft outcome following kidney transplantation consider information at the human leukocyte antigen (HLA) loci. Cell surface antigens, in addition to HLA, may serve as the stimuli as well as the targets for the anti-allograft immune response and influence long-term graft outcomes. We therefore performed exome sequencing of DNA from kidney graft recipients and their living donors and estimated all possible cell surface antigens mismatches for a given donor/recipient pair by computing the number of amino acid mismatches in trans-membrane proteins. We designated this tally as the allogenomics mismatch score (AMS). We examined the association between the AMS and post-transplant estimated glomerular filtration rate (eGFR) using mixed models, considering transplants from three independent cohorts (a total of 53 donor-recipient pairs, 106 exomes, and 239 eGFR measurements). We found that the AMS has a significant effect on eGFR (mixed model, effect size across the entire range of the score: -19.4 [-37.7, -1.1], P = 0.0042, χ2 = 8.1919, d.f. = 1) that is independent of the HLA-A, B, DR matching, donor age, and time post-transplantation. The AMS effect is consistent across the three independent cohorts studied and similar to the strong effect size of donor age. Taken together, these results show that the AMS, a novel tool to quantify amino acid mismatches in trans-membrane proteins in individual donor/recipient pair, is a strong, robust predictor of long-term graft function in kidney transplant recipients.

## Author Summary

The article describes a new concept to help match donor organs to recipients for kidney transplantation. The concept relies on the ability to measure the individual DNA of potential donors and recipients. When the data about genomes (i.e., DNA) of possible donors and recipients are available, the article describes how data can be computationally compared to identify differences in these genomes and quantify the possible future impact of these differences on the functioning of the graft. The concept presented in the article determines a score for each pair of possible donor and recipient. This score is called the allogenomics mismatch score. The study tested the ability of this score to predict graft function (the ability of the graft to filter blood) in the recipient several years after transplantation surgery. The study found that, in three small sets of patients tested, the score is a strong predictor of graft function. Prior studies often assumed that only a small number of locations in the genome were most likely to have an impact on graft function, while this study found initial evidence that differences across DNA that code for a large number of proteins can have a combined impact on graft function.

## Introduction

Survival of patients afflicted with End Stage Renal Disease (ESRD) is superior following kidney transplantation compared to dialysis therapy. The short-term outcomes of kidney grafts have steadily improved since the early transplants with refinements in immunosuppressive regimens, use of DNA-based human leukocyte antigen (HLA) typing, and better infection prophylaxis [1–3]. Despite these advances, data collected across the USA and Europe show that 40–50% of kidney allografts fail within ten years of transplantation [4]. This observation strongly suggests that as yet uncharacterized factors, including genomic loci, may adversely impact long-term post-transplantation outcomes.

The HLA is a cluster of genes on the short arm of chromosome 6 and constitutes the major histocompatibility complex (MHC) responsible for self/non-self discrimination in humans. Multiple clinical studies have demonstrated the importance of HLA-matching to improve kidney graft outcome. Therefore, in many countries, including the USA, donor kidney allocation algorithms includes consideration of HLA matching of the kidney recipient and donor. With widespread incorporation of HLA matching in kidney organ allocation decisions, it has become clearer that HLA mismatching represents an important risk factor for kidney allograft failure but fails to fully account for the invariable decline in graft function and failure in a large number of recipients over time. Indeed, only a 15% survival difference exist at 10 years post transplantation between the fully matched kidneys and the kidneys mismatched for both alleles at the HLA-A, B and DR loci [5]. Findings from large cohorts of kidney graft recipients have also been studied to separate the immunological effect mediated by HLA and the non-HLA effects [6]. Overall, prior observations suggest that mismatches at non-HLA loci in the genome could influence long-term graft outcomes. Also, antibodies directed at HLA as well as non-HLA (e.g., MHC class I polypeptide-related sequence [MICA]) have been associated with allograft rejection and reduced graft survival rates. Indeed, it has been reported that the presence of anti-MICA antibodies in the pre-transplant sera is associated with graft failure despite HLA matching of the kidney recipient with the organ donor.

Here, we used exome sequencing to determine the sequences of the HLA as well as non-HLA peptides encoded by the donor organ and displayed on its cell surface, as well as bioinformatics analyses to determine donor sequences not present in the recipient. The allogenomics approach integrates the unique features of transplantation, such as the existence of two genomes in a single individual, and the recipient’s immune system mounting an immune response directed at either HLA or non-HLA antigens displayed by the donor kidney. In this report, we show that this new concept helps predict long-term kidney transplant function from the genomic information available prior to transplantation. We found that a statistical model that incorporates time as covariate, HLA, donor age and the AMS (allogenomics mismatch score, introduced in this study), predicts graft function through time better than a model that includes the other factors and covariates, but not the AMS.

## Results

### The allogenomics concept and the allogenomics mismatch score (AMS)

**Fig 1:**
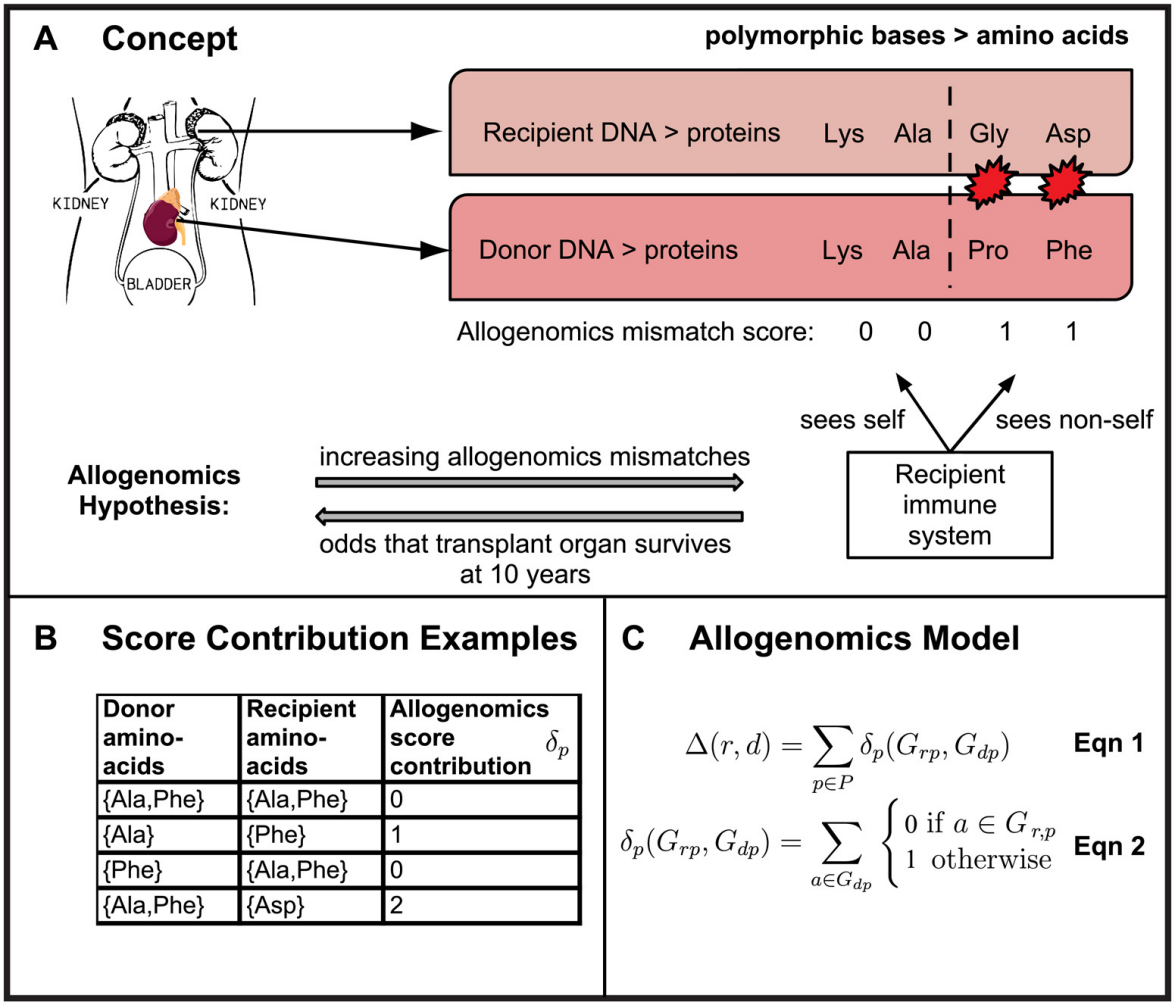
Recipient/Donor incompatibility quantified by exome sequencing and calculation of allogenomics mismatch score (AMS). **(A)** Hypothesis: Post-transplantation kidney graft function is associated with the number of amino acids coded by the donor genome that the recipient’s immune system could recognize as non-self. **(B)** Examples of donor/recipient amino-acid mismatches at one protein position, and resulting contributions to the allogenomics mismatch score. The allogenomics mismatch score is calculated by summing contributions over a set of genomic polymorphisms (see Methods for details). **(C)** Equations for the allogenomics model. Score contributions are summed across all genomic positions of interest (set *P*) to yield the allogenomics score Δ(*r*,*d*). *G*_*r*,*p*_: genotype of recipient *r* at genomic site/position *p*. *G*_*d*,*p*_: genotype of donor *d* at site *p*. Alleles of a genotype are denoted with the letter *a*.

The allogenomics concept is the hypothesis that interrogation of the coding regions of the entire genome for both the organ recipient and organ donor DNA can identify the number of incompatible amino-acids (recognized as non-self by the recipient) that inversely correlates with long-term function of the kidney allograft. Fig 1A is a schematic illustration of the allogenomics concept. Because human autosomes have two copies of each gene, we consider two possible alleles in each genome of a transplant pair. To this end, we estimate allogenomics score contributions between zero and two, depending on the number of different amino acids that the donor genome encodes for at a given protein position. Fig 1B shows the possible allogenomics score contributions when the amino acids in question are either an alanine, or a phenylalanine or an aspartate amino acid. The allogenomics mismatch score (AMS) is a sum of amino acid mismatch contributions. Each contribution represents an allele coding for a protein epitope that the donor organ may express and that the recipient immune system could recognize as non-self (see Equation 1 and 2 in Fig 1C and Materials and Methods and full description in S1 File).

We have developed and implemented a computational approach to estimate the AMS from genotypes derived for pairs of recipient and donor genomes. (See Materials and Methods for a detailed description of this approach and its software implementation, the allogenomics scoring tool, available at http://allogenomics.campagnelab.org.) Our approach was designed to consider the entire set of protein positions measured by a genotyping assay, or restrict the analysis to a subset of positions *P* in the genome. In this study, we focused on the subset of genomic sites P that encode for amino acids in trans-membrane proteins. It is possible that some secreted or intra-cellular proteins can contribute to the allogenomics response, but the set of trans-membrane proteins was considered in this study in order to enrich contributions for epitopes likely to be displayed at the surface of donor kidney cells. While proteins expressed in kidney could appear to be a better choice, the technical challenge of defining a list of proteins expressed by kidney alone, and perhaps only transiently in some kidney cell type exposed to the surface of the kidney, argues against relying on a kidney expression filter. Similarly, we did not consider other sets of proteins, and make no claim that the set of transmembrane proteins is an optimal choice.

Because the AMS sums contributions from thousands of genomic sites across the genome, it is an example of a burden test, albeit summed across an entire exome. The procedure is akin to averaging and the resulting score is much less sensitive to errors introduced by the genotyping assays or analysis approach than previous association studies which considered genotypes individually. The AMS approach yields a single score per transplant. This eliminates the need to correct for tens of thousands of statistical tests, which are common in classical association studies. The allogenomics approach therefore also decreases the number of samples needed to reach statistical power.

### Testing the association between AMS and kidney allograft function measured post-transplantation

In order to test the allogenomics hypothesis, we isolated DNA from kidney graft recipients and their living donors. We assembled three cohorts: **Discovery Cohort (10 transplant pairs)** where the allogenomics observation was first made (these patients were a subset of patients enrolled in a multicenter Clinical Trial in Organ Transplantation-04 study of urinary cell mRNA profiling, from whom tissue/cells were collected for future mechanistic studies [7], 10 transplant pairs), and two validation cohorts: one from recipients transplanted at the New York Presbyterian Weill Cornell Medical Center (**Cornell Validation Cohort**, 24 pairs), and a second validation cohort from recipients transplanted in Paris hospitals (**French Validation Cohort**, 19 pairs).

Table 1 provides demographic and clinical information about the patients included in our study. Exome data were obtained for each cohort. For the Discovery cohort, we used the Illumina TrueSeq exome enrichment kit v3, covering 62Mb of the human genome. For the two validation cohorts, DNA sequencing was performed using the Agilent Haloplex assay covering 37Mb of the coding sequence of the human genome. Primary sequence data analyses were conducted with GobyWeb [8] (data and analysis management), Last [9] (alignment to the genome) and Goby [10] (genotype calls). Table A in S1 File provides statistics of coverage for the exome assays.

**Table 1:** Characteristics of Kidney transplant recipients and their donors. In bold, characteristics that differ between the Cornell validation cohort and the French validation cohort (*P<0.05, two tailed t-test).

Characteristic	Discovery cohort	Cornell validation cohort	French validation cohort
Number of Transplant Pairs with living donors	10/10	24/24	19/19
Allogenomics mismatch score AMS(SD)[range]	1335(304)[994–2033]	1094(259)[700–1630]	560(147)[349–811]
Clinical factors			
Age			
Donor (SD)	41 (13)	46 (10)	44(16)
Recipient (SD)	48 (10)	51 (13)	38(15)
Living Donor type			
Living related N (AMS) [SD])	4 (1116 [143])	13 (939 [218])	15(503[108])*
Living unrelated N (AMS) [SD])	6 (1481 [300])	11(1277 [170])	4(769[41])*
Donor sex			
Male (%)	2 (20%)	8 (33%)	6(32%)
Female (%)	8 (80%)	16 (67%)	13(68%)
Donor Race			
Black (%)	4(40%)	5 (21%)	2(10%)
Non-Black (%)	6(60%)	19 (79%)	17(90%)
Recipient sex			
Male (%)	9 (90%)	13 (54%)	13 (53%)
Female (%)	1 (10%)	11 (46%)	13 (47%)
Recipient Race			
Black (%)	4 (40%)	7 (29%)	2 (10%)
Non-Black (%)	6 (60%)	17 (71%)	17 (90%)
Number of HLA mismatches ABDR (SD)	3.9 (1.91)	3.5 (1.89)	2.5 (1.68)*
Functional Factors			
Number of Patients at 12 months	10	24	17
Serum creatinine level at 12 months mg/dL (SD)	1.51 (0.35)	1.45 (0.41)	1.29 (0.41)
eGFR at 12 months ml/min/1.73m^2^ (SD)	54.3(10)	54.3 (16.3)	61.8 (18.9)*
Number of Patients at 24 months	9	23	19
Serum creatinine level at 24 months mg/dL (SD)	1.36 (0.19)	1.45 (0.49)	1.26 (0.3)
eGFR at 24 months ml/min/1.73m^2^ (SD	59 (7.7)	54.85 (15.7)	59.3 (14.5)*
Number of Patients at 36 months	8	22	19
Serum creatinine level at 36 months mg/dL(SD)	1.62 (0.50)	1.38 (0.40)	1.35 (0.45)
eGFR at 36 months ml/min/1.73m^2^ (SD)	53.4 (15)	55.3 (15.9)	56.3 (16.4)
Number of Patients at 48 months	0	16	16
Serum creatinine level at 48 months mg/dL(SD)	-	1.34 (0.43)	1.40 (0.56)
eGFR at 48 months ml/min/1.73m^2^ months (SD)	-	57.4 (16.4)	55.7 (18.2)
Patients with an Acute Cellular rejection episode in the first year of transplantation, N (%)	3 (30%)	5 (20%)	2 (10%)
Immunosupression			
Calcineurin Inhibitors, n (%)	9 (90%)	24 (100%)	19 (100%)
Corticosteroids, n (%)	0 (0%)	5 (21%)	17 (90%)*

Kidney graft function is a continuous phenotype and is clinically evaluated by measuring serum creatinine levels or using estimated glomerular filtration rate (eGFR) [11]. In this study, kidney graft function was evaluated at several time points for each recipient, with the precise time points varying by cohort. In the discovery cohort, kidney allograft function was measured at 12, 24, 36 and 48 months following transplantation using serum creatinine levels and eGFR, calculated using the 2011 MDRD [11] formula. We examined whether the allogenomics mismatch score is associated with post-transplantation allograft function.

**Fig 2:**
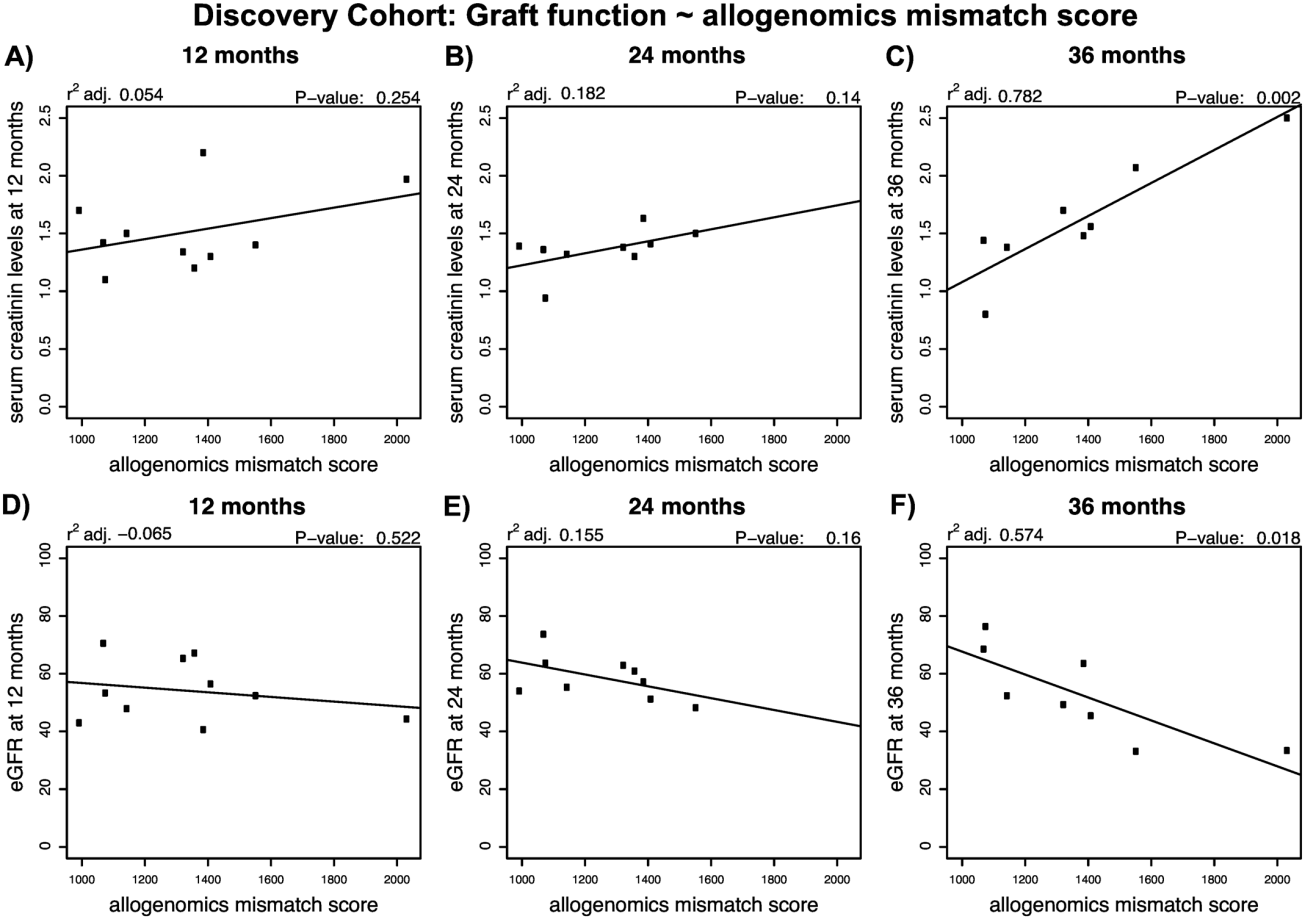
Relationship between the allogenomics mismatch score (AMS) and kidney graft function at 12, 24 or 36 months following transplantation in the Discovery cohort. DNA was isolated from 10 pairs of kidney graft recipients and their living kidney donors (Discovery set). Whole exome sequencing of the donor genomes and recipient genomes was performed and the sequencing information was used to calculate allogenomics mismatch scores based on amino acid mismatches in trans-membrane proteins. The panels depict the relationship between the allogenomics mismatch scores and serum creatinine levels at 12, 24 and 36 months post transplantation (Panels A, B and C, respectively) and the relationship between the allogenomics mismatch scores and estimated glomerular filtration rate at 12, 24 and 36 months post transplantation (Panels D, E and F, respectively). Both serum creatinine levels and eGFR correlate in a time-dependent fashion with the allogenomics mismatch score with the strongest correlations being observed at 36 months post-transplantation.

In Fig 2, we illustrate the association observed between AMS and creatinine levels or eGFR in the Discovery Cohort. We found positive linear associations between the allogenomics mismatch score and serum creatinine level at 36 months post transplantation (r^2^ adj. = 0.78, P = 0.002, n = 10) but not at 12 or 24 months following kidney transplantation (Fig 2A, 2B and 2C). We also found a negative linear relationship between the score and eGFR at 36 months post transplantation (r^2^ adj. = 0.57, P = 0.02) but not at 12 or 24 months following kidney transplantation (Fig 2D, 2E and 2F). These findings suggest that in the Discovery cohort the AMS is predictive of long-term graft function. It is also possible that the AMS score would predict short-term graft function, but that more data is needed to detect smaller changes in eGFR at early time points, whereas cumulative effects on graft function become detectable at later time points. Similar observations were made in the two validation cohorts (see Figures A and B in S1 File) and discussed in detail in an earlier preprint [12].

### The AMS predicts graft function longitudinally across three independent cohorts

In the models presented so far, we have considered the prediction of graft function separately at different time points. An alternative analysis would consider time since transplantation, as well as other established predictors of graft function as covariates in the model. This is particularly useful when studying cohorts where graft function was assessed at several distinct time points (e.g., in the French cohort, clinical data describes graft function from 1 to 96 months post transplantation, but few time points have observations for all recipients). To implement this alternative analysis, we fit a mixed linear model of the form: eGFR ~ donor age at time of transplant + AMS + T + (1|P) (**Equation 3**), where T is the time post-transplantation, measured in months, and (1|P) a random effect which models separate model intercepts for each donor/recipient pairs.

To determine the effect of AMS on eGFR, we compared the fit of models that did or did not include the AMS. We found that the effect of AMS is significant (P = 0.0042, χ2 = 8.1919, d.f. = 1). A similar result was obtained if HLA was also used as a covariate in the model (i.e., eGFR ~ donor age at time of transplant + AMS + T + HLA + (1|P) (**Equation 4**), comparing model with AMS or without, P = 0.038, χ2 = 4.284, d.f. = 1). In contrast, models that included AMS, but did or did not include the number of ABDR HLA mismatches fit the data equally well (testing the effect of HLA, P = 0.60, χ2 = 0.2737, d.f. = 1), confirming that the effect of AMS was independent of the number of HLA mismatches. The models of equations 3 and 4 include a random effect for the transplant pair (1|P) term. This term models the differences among pairs, such as level of graft function in the days post-transplantation, as well as correlations between repeated measurements for the same recipient. See Fig C in S1 File for a more direct comparison between AMS and HLA ABDR mismatches. This comparison indicates that there is a moderate correlation between AMS and the number of HLA ABDR mismatches. Taken together, these results indicate that the predictive ability of the AMS effect is mostly independent of the number of ABDR mismatches at the HLA loci.

In order to determine if the AMS effect is robust, we fit the model from equation 3 in each cohort independently. The estimates for the AMS effect are shown in Table 2. Despite a limited amount of data to fit the model in each cohort, the estimates are very similar, strongly suggesting that the AMS effect is robust and can be observed even in small cohorts (10, 19 and 24 transplant pairs).

**Table 2:** Robustness of the AMS effect across cohorts.

Cohort	# Transplant Pairs	# eGFR Observations	AMS effect size (from Eq. 3)
Discovery	10	27	-0.01994
Cornell Validation	24	90	-0.01748
French Validation	19	122	-0.01844
Combined	53	239	-0.01307

In Fig D in S1 File we plot the minor allele frequencies (MAF) of the variations that contribute to the AMS in the Discovery and Validation cohorts. We find that many polymorphisms that contribute to the AMS have low MAF, indicating that they are rare in human populations. This point needs to be considered for replication studies. For instance, GWAS genotyping platforms may require adequate imputation to infer polymorphisms with low MAF.

Table 3 presents confidence intervals for the parameters of the full model (equation 4, including HLA term), fit across 53 transplant pairs, as well as the effective range of each of the model predictors. The table shows the expected impact of each predictor on eGFR when this predictor is varied over its range, assuming all other predictors are kept constant. For instance, assume that donor age at time of transplant varies from 20 years old to 80 years old (range: 60). Across this range, eGFR will decrease by an estimated 28 units as the donor gets older. The AMS effect has an effective range of 1,700 and the corresponding eGFR decrease is 19 units. This comparison indicates that the strength of the AMS effect is similar to that of donor age and more than five times larger than the effect of HLA- ABDR mismatches.

**Table 3:** Estimated model parameters, 95% confidence intervals and expected impact on eGFR.

		Estimate			Impact on eGFR			
Model Coefficient	Fit	2.50%	97.50%	Effective Range	Fit	2.50%	97.50%	Note
Time post transplantation (in months)	-0.24	-0.32	-0.16	480	-117.07	-155.27	-78.95	*
Donor age at transplant (in years)	-0.47	-0.78	-0.15	60	-28.11	-46.86	-9.11	*
AMS	-0.01	-0.022	-0.00063	1700	-19.40	-37.69	-1.07	*
HLA- ABDR mismatches	-0.57	-2.74	1.61	6	-3.42	-16.42	9.64	**n.s.**

## Discussion

While HLA-matching is a necessary requirement for successful hematopoietic cell transplants, full HLA compatibility is not an absolute prerequisite for all types of transplantations as indicated by the thousands of solid organ transplants performed yearly despite lack of full matching between the donor and recipient at the HLA-A, B and DR loci. In view of better patient survival following transplantation compared to dialysis, kidney transplants have become the standard of care for patients with end stage kidney disease and transplants are routinely performed with varying degrees of HLA-class I and II mismatches. Although, graft outcomes improve with better HLA-matching [13], excellent long-term graft outcomes with stable graft function have been observed in patients with full HLA -ABDR mismatches. The success of these transplants clearly suggests that factors other than HLA compatibility may influence the long-term clinical outcome of kidney allografts. Furthermore, grafts do fail even with the best HLA match [13], suggesting that antigens other than HLA are targets of alloimmune response. Indeed, several non-HLA antibodies have been identified for renal and cardiac allograft recipients and found detrimental to long-term outcome [14,15]. These antibodies were found to target antigens expressed on endothelial and epithelial cells but also on a variety of parenchymal and immune cells and can be measured prior to transplantation. These prior studies support the notion that non-HLA antibodies can influence long-term outcome in transplantation.

Recipients of a kidney transplant have two genomes in their body: their germline DNA, and the DNA of the donor. It is clear that a Mendelian genetic transmission mechanism is not at play in transplantation, yet, this assumption has been made in most of the transplantation genomic studies published to date [16,17]. While several case-control studies have been conducted with large organ transplant cohorts, the identification of genotype/phenotype associations has been limited to the discoveries of polymorphisms with small effect, that have been reviewed in [18], and have often not been replicated [19–21]. Rather than focusing on specific genomic sites, the allogenomics concept sums contributions of many mismatches that can impact protein sequence and structure and could engender an immune response in the graft recipient. These allogenomics mismatches, captured in our study, represent the sequences of non-HLA trans-membrane proteins, some of which may help initiate cellular and humoral immunity directed at the allograft.

This study used eGFR as a surrogate marker for long-term graft survival. The advantage of focusing on eGFR is that it is measured as part of clinical care on a yearly basis for each recipient, and eGFR has been associated with long-term outcome in multiple studies. Since acute rejection has also been associated with a decrease in long-term graft survival, it may also serve as a surrogate marker for long-term kidney allograft survival. Acute rejection however is a rare event with current immunosuppressive regimens and given the relatively small size of our study cohort, we would not have had sufficient cases to examine the association between acute rejection and the allogenomics score. Another consideration for not using acute rejection is that acute rejection only represents a fraction of the mechanisms that lead to graft loss [22].

The allogenomics concept that we present in this manuscript postulates a mechanism for the development of the immune response in the transplant recipient: immunological and biophysical principles strongly suggest that alleles present in the donor genome, but not in the recipient genome, will have the potential to produce epitopes that the recipient immune system will recognize as non-self. This reasoning explains why the allogenomics score is not equivalent to the genetic measures of allele sharing distance that have been used to perform genetic clustering of individuals [23]. This manuscript also suggests that allogenomic mismatches in proteins expressed at the surface of donor cells could explain why some recipients’ immune systems mount an attack against the donor organ, while other patients tolerate the transplant for many years, when given similar immunosuppressive regimens. If the results of this study are confirmed in additional independent transplant cohorts (renal transplants, solid or hematopoeitic cell transplants), they may prompt the design of prospective clinical trials to evaluate whether allocating organs to recipients with a combination of low allogenomics mismatch scores and different HLA mismatch scores improves long term graft outcome. A positive answer to this question could profoundly impact the current clinical and regulatory framework for assigning organs to ESRD patients.

In this study, we introduced the allogenomics concept to quantitatively estimate the histoincompatibility between living donor and recipient outside of the HLA loci. We tested the simplest model derived from this concept to calculate an allogenomics mismatch score (AMS) reflecting the possible donor specific epitopes displayed on the cell surface. We demonstrated that the AMS, which can be estimated before transplantation, helps predict post-transplantation kidney graft function more accurately than HLA-mismatches alone. Interestingly, the strength of the correlation increases with the time post transplantation, an intriguing finding observed in both the discovery cohort and the validation cohorts.

We chose the simplest model to test the allogenomics concept and did not restrict the score to contributions from the peptides that can fit in the HLA groove despite their computational predictability [24]. It is possible that such restriction would increase the score’s ability to predict renal function post transplantation. However, such a filter assumes that HLA and associated peptides are the only stimuli for the anti-allograft response and does not take into consideration allorecognition involving innate effectors (NK cells or NKT cells for example, the Killer-cell Immunoglobulin-like Receptor KIR genes, iTCR, the invariant T Cell Receptor, and TLR, Toll Like Receptor, among others) [25]. The allogenomics concept incorporating amino acid mismatches capable of triggering adaptive as well as innate immunity could be considered an important strength of the approach.

Recent evidence indicates that mutations in splice sites, although rare, are responsible for a large proportion of disease risk [26]. The allogenomics approach presented in this manuscript does not incorporate knowledge of how polymorphisms in splice sites affect protein sequences. We anticipate that future developments would consider longer splice forms in the donor as allogenomics. Such an approach could score additional donor protein residues as allogenomics mismatches when the sequence is not present in the predicted proteome of the recipient.

We chose to focus this study on living, ABO compatible (either related or non-related) donors because kidney transplantation can be planned in advance and because differences in cold ischemia times and other covariates common in deceased donor transplants are negligible when focusing on living donors, especially in small cohorts. The selection criteria for deceased donors include consideration of HLA matching, calculated panel reactive antibody and the age of the recipient. Compared to live donors we expect that the range of the AMS in deceased donors will be comparable to that in our discovery cohort composed primarily of unrelated donors. Since many additional factors can independently influence graft function after transplantation from a deceased donor (e.g. cold ischemia time), potentially much larger cohorts may be required in such settings to achieve sufficient power to adequately control for the covariates relevant to deceased donors and to detect the allogenomics effect.

While we have not attempted to optimize the set of sites considered to estimate the allogenomics mismatch score, it is possible that a reduced and more focused subsets of amino acid mismatches could increase the predictive ability of the score. For instance, the AMS could be applied to look for genes with a high allogenomic mismatch burden. Such studies would require larger cohorts and may enable the discovery of loci enriched in allogenomics mismatches responsible for a part of the recipient alloresponse against yet unsuspected donor antigens. Their discovery might foster the development of new immunosuppressive agents targeting the expression of these immuno-dominant epitopes. However, our study also raises a novel mechanistic hypothesis: the total burden of allogenomics mismatches might be more predictive of graft function, than mismatches at specific loci, as was previously widely expected [17].

## Materials and Methods

### Ethics statement

The study was reviewed and approved by the Weill Cornell Medical College Institutional Review Board (protocol #1407015307 “Predicting Long-Term Function of Kidney Allograft by Allogenomics Score”, approved 09/09/2014). The second study involving the French cohort was approved by the Comité de Protection des Personnes (CPP), Ile de France 5, (02/09/2014). Codes were used to ensure donor and recipient anonymity. All subjects gave written informed consent. Living donor ABO compatible kidney transplantations were performed according to common immunological rules for kidney transplantation with a mandatory negative IgG T-cell complement-dependent cytotoxicity cross-match.

### Whole exome sequencing and genotyping

Briefly, genotypes of donors and recipients were assayed by exome sequencing (Illumina TruSeq enrichment kit for the Discovery Cohort and Agilent Haloplex kit for the Cornell Validation Cohort and the French Validation Cohort). Reads were aligned to the human genome with the Last [9] aligner integrated as a plugin in GobyWeb [8]. Genotype calls were made with Goby [10] and GobyWeb [8]. Prediction of polymorphism impact on the protein sequence were performed with the Variant Effect Predictor [27]. Genes that contain at least one transmembrane segment were identified using Ensembl Biomart [28].

#### HLA typing

The HLA genotypes were obtained from the clinical information system. HLA genotypes are performed as part of clinical care in transplantation centers. They are conducted with the SSP and SSO methods[29].

### Discovery cohort: Transplant recipients and DNA samples

We selected 10 kidney transplant recipients from those who had consented to participate in the Clinical Trials in Organ Transplantation-04 (CTOT-04), a multicenter observational study of noninvasive diagnosis of renal allograft rejection by urinary cell mRNA profiling. We included only the recipients who had a living donor kidney transplant and along with their donors, had provided informed consent for the use of their stored biological specimens for future research. Pairs were limited to those where enough DNA could be extracted to perform the exome assay for both donor and recipient. Subjects were not selected on the basis of eGFR, whose values were collected after obtaining sequence data. The demographic and clinical information of the Discovery cohort is shown in Table 1. DNA was extracted from stored peripheral blood using the EZ1 DNA blood kit (Qiagen) based on the manufacturer’s recommendation.

### Discovery cohort: Whole exome sequencing

DNA was enriched for exome regions with the TruSeq exome enrichment kit v3. Sequencing libraries were constructed using the Illumina TruSeq kit DNA sample preparation kit. Briefly, 1.8 μg of genomic DNA was sheared to average fragment size of 200 bp using the Covaris E220 (Covaris, Woburn, MA, USA). Fragments were purified using AmpPureXP beads (Beckman Coulter, Brae, CA, USA) to remove small products (<100 bp), yielding 1 μg of material that was end-polished, A-tailed and adapter ligated according to the manufacturer’s protocol. Libraries were subjected to minimal PCR cycling and quantified using the Agilent High Sensitivity DNA assay (Agilent, Santa Clara, CA, USA). Libraries were combined into pools of six for solution phase hybridization using the Illumina (Illumina, San Diego, CA, USA) TruSeq Exome Enrichment Kit. Captured libraries were assessed for both quality and yield using the Agilent High Sensitivity DNA assay Library Quantification Kit. Sequencing was performed with six samples per lane using the Illumina HiSeq 2000 sequencer and version 2 of the sequencing-by-synthesis reagents to generate 100 bp single-end reads (1×100SE).

### Cornell validation cohort: Transplant recipients, donors and DNA samples

We studied 24 kidney transplant recipients who had a living donor transplant at the NewYork-Presbyterian Weill Cornell Medical Center. This was an independent cohort and none of the recipients had participated in the CTOT-04 trial. Recipients were selected randomly based on the availability of archived paired recipient-donor DNA specimens obtained at the time of transplantation at our Immunogenetics and Transplantation Laboratory. DNA extraction from peripheral blood was done using the EZ1 DNA blood kit (Qiagen) based on the manufacturer’s recommendation.

### French validation cohort: Transplant recipients, donors and DNA samples

We studied 19 kidney transplant recipients who had a living donor transplant at Tenon Hospital. This represented a third independent cohort. Recipients were selected randomly based on the availability of archived paired recipient-donor DNA specimens obtained either at the Laboratoire d'histocompatibilité, Hôpital Saint Louis APHP, Paris or during patient’s follow-up between October 2014 and January 2015. DNA extraction from peripheral blood was done using the Nucleospin blood L kit (Macherey-Nagel) based on the manufacturer’s recommendation.

### Cornell and french validation cohorts: Whole exome sequencing

The Cornell and French Validation cohorts were both assayed with the Agilent Haloplex exome sequencing assay. The Haloplex assay enriches 37 Mb of coding sequence in the human genome and was selected for the validation cohort because it provides a strong and consistent exome enrichment efficiency for regions of the genome most likely to contribute to the allogenomics contributions in protein sequences. In contrast, the TrueSeq assay (used for the Discovery Cohort) enriches 63Mb of sequence and includes regions in untranslated regions (5’ and 3’ UTRs), which do not contribute to allogenomics scores and therefore do not need to be sequenced to estimate the score. Libraries were prepared as per the Agilent recommended protocol. Sequencing was performed on an Illumina 2500 sequencer with the 100bp paired-end protocol recommended by Agilent for the Haloplex assay. Libraries were multiplexed 6 per lane to yield approximately 30 million paired end reads per sample.

### Minor allele frequencies of the AMS sites

We determined the minor allele frequency of sites used in the calculation of the allogenomics mismatch score using data from the Exome Aggregation Consortium (ExAC). This resource made it possible to estimate MAF for most of the variations that are observed in the subjects included in our discovery and validation cohort. Data was downloaded and analyzed with R and MetaR scripts (see analysis scripts provided at https://bitbucket.org/campagnelaboratory/allogenomicsanalyses).

### Overlap with ESP variants

We use the NHLBI Exome Sequencing Project (ESP) release ESP6500SI-V2 [30]. The ESP measured genotypes in a population of 6,503 individuals across the EA and AA populations using an exome-sequencing assay [30]. Of 12,657 sites measured in the validation cohort with an allogenomics contribution strictly larger than zero (48 exomes, sites with contributions across 24 clinical pairs of transplants), 9,765 (78%) have also been reported in ESP (6,503 exomes).

### Sequence data analysis

Illumina sequence base calling was performed at the Weill Cornell Genomics Core Facility. Sequence data in FASTQ format were converted to the compact-reads format using the Goby framework [14]. Compact-reads were uploaded to the GobyWeb[8] system and aligned to the 1000 genome reference build for the human genome (corresponding to hg19, released in February 2009) using the Last [9,31] aligner (parallelized in a GobyWeb [8] plugin). Single nucleotide polymorphisms (SNPs) and small indels genotype were called using GobyWeb with the Goby [32] discover-sequence-variants mode (parameters: minimum variation support = 3, minimum number of distinct read indices = 3) and annotated using the Variant Effect Predictor [27] (VEP version 75–75.7) from Ensembl. The data were downloaded as a Variant Calling format [33] (VCF) file from GobyWeb [8] and further processed with the allogenomics scoring tool (see http://allogenomics.campagnelab.org).

### Estimation of the Allogenomics Mismatch Score (AMS) and allogenomics tools

The allogenomics mismatch score Δ(*r*,*d*) is estimated for a recipient *r* and donor *d* as the sum of score mismatch contributions (see Fig 1C and **supplementary methods in** S1 File).

### Statistical analyses

Analyses were conducted with either JMP Pro version 11 (SAS Inc.) or metaR (http://metaR.campagnelab.org). Fig 2 as well as Figures in S1 File were constructed with metaR analysis scripts and edited with Illustrator CS6 to increase some font sizes or adjust the text of some axis labels. The model that includes the time post-transplantation as a covariate was constructed in metaR and JMP. The R implementation of train linear model uses the lm R function. This model was executed using the R language 3.1.3 (2015-03-09) packaged in the docker image fac2003/rocker-metar:1.4.0 (https://hub.docker.com/r/fac2003/rocker-metar/). Models with random effects were estimated with metaR 1.5.1 and R (train mixed model and compare mixed models statements, which use the lme4 R package [34]). Comparison of fit for models with random effects was obtained by training each model alternative with REML = FALSE an performing an anova test, as described in [35]. We distribute the code necessary to reproduce most of the analysis presented in this manuscript at https://bitbucket.org/campagnelaboratory/allogenomicsanalyses.

## Supporting Information

S1 File Supplementary Table, Methods, Results and Figures. This file provides additional details about method descriptions, additional results, as well as supplementary Table and Figures.
(PDF)
