# Supplementary material for: Exome Sequencing and Prediction of Long-Term Kidney Allograft Function
Source: PLoS Comput Biol. 2016 Sep 29;12(9):e1005088. doi: 10.1371/journal.pcbi.1005088 (PMC5042552; doi:10.1371/journal.pcbi.1005088)
Supplement: S1 File — This file provides additional details about method descriptions, additional results, as well as supplementary Table and Figures. (PDF) [file pcbi.1005088.s001.pdf]

Exome Sequencing and Prediction of Long-Term Kidney Allograft Function

Supplementary Table, Methods, Results and Figures

Authors: Laurent Mesnard[1,7,9], Thangamani Muthukumar[2,3], Maren Burbach[2], Carol Li[2], Huimin Shang[4], Darshana Dadhania[2,3], John R. Lee[2,3], Vijay K. Sharma[2], Jenny Xiang[4] , Caroline Suberbielle[8] , Maryvonnick Carmagnat[8], Nacera Ouali[7], Eric Rondeau[7,9], John J. Friedewald[5], Michael M. Abecassis[6], Manikkam Suthanthiran*[2,3], Fabien Campagne*[1]

Affiliations:

[1] The HRH Prince Alwaleed Bin Talal Bin Abdulaziz Alsaud Institute for Computational Biomedicine, Weill Cornell Medical College, New York, NY, United States of America; Department of Physiology and Biophysics, The Weill Cornell Medical College, New York, NY, United States of America

[2] Division of Nephrology and Hypertension, Weill Cornell Medical College, New York, NY, United States of America

[3] Department of Transplantation Medicine, New York Presbyterian Hospital, New York, NY, United States of America

[4] Genomics Core Facility Weill Cornell Medical College

[5] Northwestern University Feinberg School of Medicine, Chicago, IL, United States of America

[6] Comprehensive Transplant Center, Northwestern University Feinberg School of Medicine, Chicago, IL, United States of America

[7] INSERM UMR1155 et Service des Urgences Néphrologiques et Transplantation Rénale, APHP, Hôpital Tenon, 75020 Paris, France

[8] Laboratoire d'histocompatibilité Hôpital Saint Louis APHP, Paris, France

[9] Sorbonne Universités, UPMC Université Paris 06, France

*To whom correspondence should be addressed: Fabien Campagne, Ph.D. (fac2003@campagnelab.org), Manikkam Suthanthiran, M.D, PhD (msuthan@med.cornell.edu).

Table A. Exome Assay Coverage Statistics

Cohort	# pairs	# exomes	Mean Coverage	% ≥10x	% ≥20x	% ≥30x	kit used	sequence targeted	genes targeted
CTOT4 (discovery)	10	20	19.5(3.4)	67.5 (7.8)	37.5 (8.2)	19 (7.25)	Truseq V3	64 MB	21 803
Cornell (validation)	24	48	30 (3.5)	81 (1.6)	64.4 (3)	51.4 (4.3)	Haloplex	37 MB	21 522
French	19	38	39.5 (3.6)	77 (5.4)	59.6 (5.8)	47 (5.1)	Haloplex	37 MB	21 522

The allogenomics mismatch score is estimated as given by Equation E1:

Equation E1 (reproduced from Equation 1, Fig 1C).

$$\Delta(r,d) = \sum_{p \in P} \delta_p(G_{rp}, G_{dp})$$

The AMS is a sum of contributions. Contributions are observed for each polymorphic site *p* in a set *P*, where *P* is determined by the genotyping assay and analysis methods, and can be further restricted (*e.g.*, to polymorphisms within genes that code for membrane proteins). Score mismatch $\delta_p(G_{rp}, G_{dp})$ contributions are calculated using the recipient genotype G_{rp} and the donor genotype G_{dp} at the polymorphic site *p*. Here, we consider that a genotype can be represented as a set of alleles that were called in a given genome. For instance, if a subject has two alleles at one polymorphic site, and we denote each allele A or B, the genotype at *p* is represented by the set {A,B}. This representation is general and sufficient to process polymorphic sites with single nucleotide polymorphisms or insertion/deletions.

Equation E2 describes how the individual score mismatch contributions are calculated at a polymorphic site of interest:

Equation E2 (reproduced from Equation 2, Fig 1C).

$$\delta_p(G_{rp}, G_{dp}) = \sum_{a \in G_{dp}} \begin{cases} 0 \text{ if } a \in G_{r,p} \\ 1 \text{ otherwise} \end{cases}$$

A contribution of 1 is added to the score for each polymorphic site where the donor genome has an allele (a_{dp}) that is not also present in the recipient genome. When both donor and recipient genomes are called at polymorphic site P, no contribution is added. For example, assume a genomic site where the donor genome has two alleles, *i.e.*, G_{dp}={A,B}, and the recipient genome is homozygote with G_{rp}={A}. In this case, (G_{rp},G_{dp})=1. Fig 1B presents additional examples of donor and recipient genotypes and indicates the resulting score contribution (the subscript *p* is omitted for conciseness). Score contributions are summed across all polymorphism sites in the set *P* to yield the allogenomic mismatch score (Equation E1).

Selection of Informative Polymorphisms

The selection of the set of polymorphic sites *P* is important to the effectiveness of the approach. In the current method, we select exonic polymorphic sites that are (1) predicted to create non-synonymous change in a protein sequence, (2) are located in a gene that codes for one or more membrane proteins (defined as any protein with at least one predicted transmembrane segment, information obtained from Biomart (23), Ensembl database 75). Additional filters can be applied to restrict *P*, which may lead to improved prediction of transplant clinical endpoints. Constructing additional filters will require the

study of a larger training set of matched recipient and donor genotypes, which currently does not exist. It is possible that such study will indicate that other criteria than (2) also lead to predictive scores.

Implementation: the Allogenomics Scoring Tool

We developed the *allogenomics scoring tool* to process genotypes in the VCF format and produce allogenomics mismatch scores for specific pairs of genomes in the input file. The *allogenomics scoring tool* was implemented in Java with the Goby framework and is designed to read VCF files produced by Goby and GobyWeb. The source code of the allogenomics scoring tool is distributed for academic and non-commercial purposes at http://allogenomics.campagnelab.org. The following command line arguments were used to generate the estimates described in this manuscript. The genotype input file(s) necessary to reproduce these results (GobyWeb tags: JEOHQUR (2.3GB), YOOLWXH (83MB)) cannot be distributed through dbGAP (http://www.ncbi.nlm.nih.gov/gap), or an equivalent archive, because the consent form signed by the CTOT-04 participants is not compatible with such distribution of the subject information.

Pre-requisite to running the command lines: (1) You must have the Java runtime environment installed on your computer (the software has been tested with version 1.6) (2) You must define the environment variable ALLO to the location where you have downloaded the distribution of the allogemomics scoring tool (3). You must obtain the input VCF files and place them under: ${ALLO}/VCF_files_input/JEOHQUR-stats.vcf.gz ${ALLO}/VCF_files_input/YOOLWXH-stats.vcf.gz.

Estimating allogenomics mismatch scores on the Discovery cohort:

```
java -Xmx4g -jar allogenomics-1.1.7-scoring-tool.jar \
        --input ${ALLO}/VCF_files_input/JEOHQUR-stats.vcf.gz \
        -p ${ALLO}/Pair_files/Discovery_cohort.pairs.tsv \
        -a Annotation_files/All_protein_coding_Ensembl_75.gtf \
        --output ${ALLO}/Output/TM-Discovery.tsv \
        --output-format TSV --only-non-synonymous-coding --vep \
        --consider-indels --minimum-depth 10 --max-depth 500 \
        -t ${ALLO}/Annotation_files/TrM-Transcript_Ensembl_75.tsv \
        --clinical
```

Estimating allogenomics mismatch scores on the Validation cohort:

```
java -Xmx4g -jar allogenomics-1.1.7-scoring-tool.jar \
        --input ${ALLO}/VCF_files_input/JEOHQUR-stats.vcf.gz \
        -p ${ALLO}/Pair_files/Validation_cohort.pairs.tsv \
        -a ${ALLO}/Annotation_files/All_protein_coding_Ensembl_75.gtf \
        --output ${ALLO}/Output/TM-Validation.tsv \
        --output-format TSV --only-non-synonymous-coding --vep \
```

```
    --consider-indels --minimum-depth 10 --max-depth 500 \
    -t ${ALLO}/Annotation_files/TrM-Transcript_Ensembl_75.tsv \
    --clinical --measured-sites SitesHaloplexExome.tsv
```

<u>Estimating allogenomics mismatch scores on the French cohort:</u>

```
java -Xmx4g -jar allogenomics-1.1.7-scoring-tool.jar \
    --input ${ALLO}/VCF_files_input/YOOLWXH-stats.vcf.gz \
    -p Pair_files/French_cohort.pairs.tsv \
    -a ${ALLO}/Annotation_files/All_protein_coding_Ensembl_75.gtf  \
    --output ${ALLO}/Output/TM-French_cohort.tsv \
    --output-format TSV --only-non-synonymous-coding \
    --vep --consider-indels --minimum-depth 10 \
    --max-depth 500 \
    -t ${ALLO}/Annotation_files/TrM-Transcript_Ensembl_75.tsv --clinical \
    --no-dash
```

<u>Estimating allogenomics mismatch scores on merged discovery and validation cohorts:</u>

```
java -Xmx4g -jar allogenomics-1.1.7-scoring-tool.jar \
    --input ${ALLO}/VCF_files_input/JEOHQUR-stats.vcf.gz\
    -p Pair_files/Discovery+Validation_cohort.pairs.tsv \
    -a ${ALLO}/Annotation_files/All_protein_coding_Ensembl_75.gtf \
    --output ${ALLO}/Output/TM-Discovery+Validation.tsv \
    --output-format TSV --only-non-synonymous-coding \
    --vep --consider-indels --minimum-depth 10 \
```

```
        --max-depth 500 \
        -t ${ALLO}/Annotation_files/TrM-Transcript_Ensembl_75.tsv --clinical
```

Estimating allogenomics mismatch score limited to Illumina GeneChip660W loci on the validation cohort:

```
java -Xmx4g -jar allogenomics-1.1.7-scoring-tool.jar \
        --input ${ALLO}/VCF_files_input/JEOHQUR-stats.vcf.gz \
        -p ${ALLO}/Pair_files/Validation_cohort.pairs.tsv \
-a ${ALLO}/Annotation_for_660W/Human660W_Gene_Annotation_hg19-ilmn.tsv \
        --output ${ALLO}/Output/TM-Validation_Illumina660W.tsv \
        --output-format TSV --only-non-synonymous-coding --vep \
        --consider-indels --minimum-depth 10 --max-depth 500 \
        -t ${ALLO}/Annotation_for_660W/TM-as-gene-names_for_Illumina660W.tsv \
        --clinical --measured-sites sites-660W.tsv
```

Supplementary Results

Impact of genotyping platform on the estimation of the AMS

We studied the impact of the genotyping platform on the estimation of the AMS (**Fig. S3**). Large cohorts of matched recipient and donor DNA are being assembled and genotyped with SNP chip array technology such as the Illumina 660W bead array platform(30). We asked whether such platforms would be appropriate to validate the allogenomics model in large cohorts. **Fig. S3A** documents the number of sites that

contribute to the allogenomics score on each platform. **Fig. S3B** indicates that the exome assay captures many more sites with rare polymorphisms (minor allele frequency <5%) than the GWAS array platform. This is expected because exome assays directly sequence an individual DNA, while GWAS platforms are designed with a fixed set of polymorphisms and will not include many of the rare polymorphisms any given individual may carry. **Fig. S3C** compares the correlations measured with the exome assay or that could have been obtained if we had measured the allogenomics mismatch score with the Illumina 660W assay. The weak correlations obtained suggest that GWAS platforms, if used without imputation, are not ideal for future tests of the allogenomics model.

Supplementary Figures are provided on the following pages.

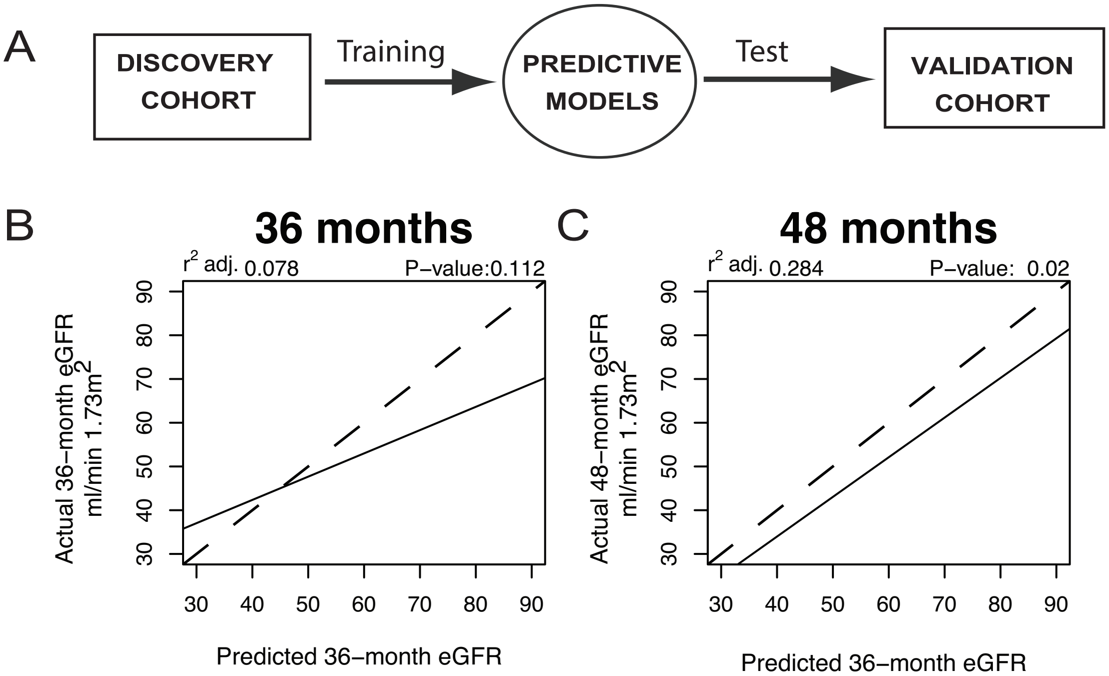

Figure A. Model trained on the Discovery cohort applied to the Validation cohort. Panel **A**) We trained a model to predict eGFR on the discovery cohort (using eGFR at 36 months) and used the trained, fixed, model to predict eGFR at 36 months and 48 months for recipients of the Validation cohort. The trained model was eGFR= 107.39547- -0.03974*AMS. Correlation between predicted eGFR and observed eGFR on the Validation cohort at 36 (Panel **B**) and 48 (Panel **C**) months post transplantation. Dashed lines indicate the diagonal and solid lines the regression lines.

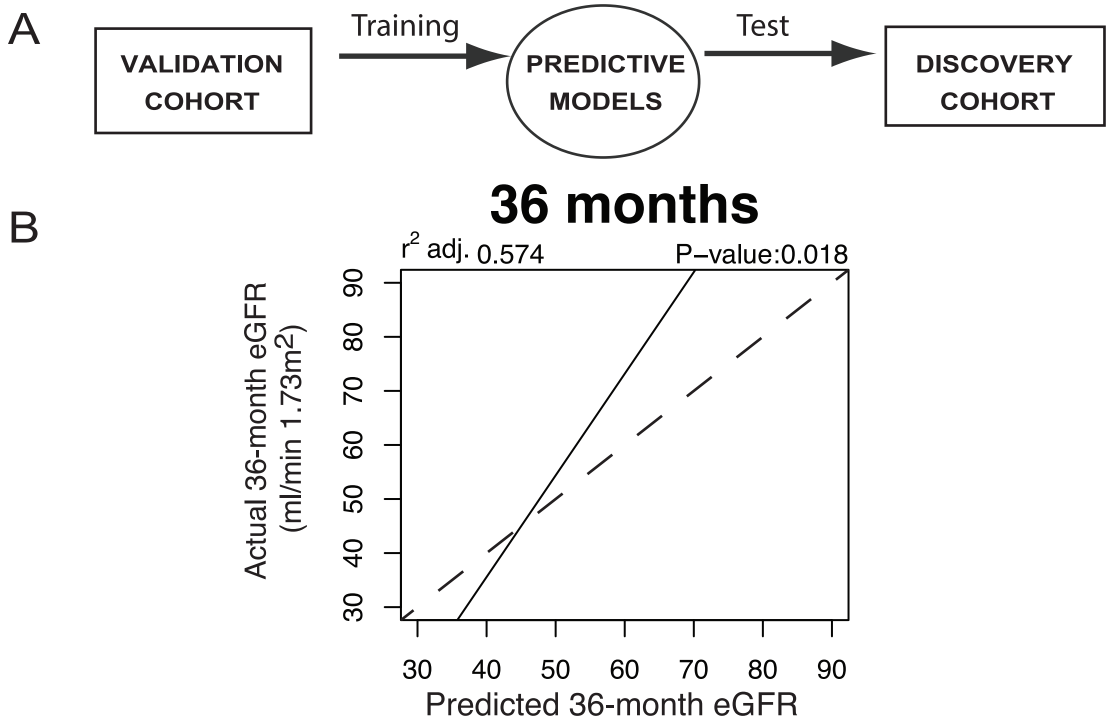

Figure B. Model trained on the Validation cohort applied to the Discovery cohort. Panel **A**) We trained models to predict serum creatinine and eGFR on the validation cohort and used the trained, fixed, model to predict serum creatinine and eGFR for recipients of the Discovery cohort. Panel **B)** Correlation between the eGFR predicted by the fixed model and that observed in the Discovery cohort. The trained model was eGFR= 78.20459 -0.02114*AMS. Dashed line indicates the diagonal and solid line the regression lines.

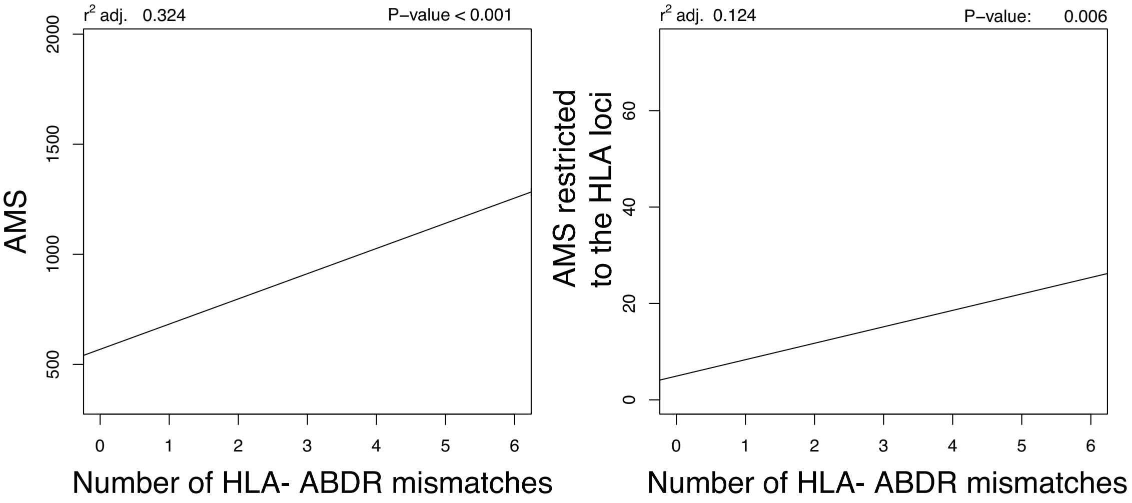

Figure C. Correlations between the AMS and HLA ABDR mismatches. Panel **A)** a moderate correlation is observed between the AMS and HLA ABDR mismatches (r^2=0.324). Note that pairs with 0 HLA ABDR mismatches have a range of AMS scores from 500-1200 units across the cohorts studied. Panel **B)** When the AMS is restricted to the mismatches that occur in the HLA genes, the correlation is weaker (r^2=0.124), illustrating that the AMS is not a simple proxy for HLA ABDR mismatches (which are counted as part of the calculation of the AMS). The relative size of the contributions can be seen by comparing the y axes across panels, with HLA ABDR mismatches contributing at most 80 units of the AMS (4-16% of the AMS values in Panel A).

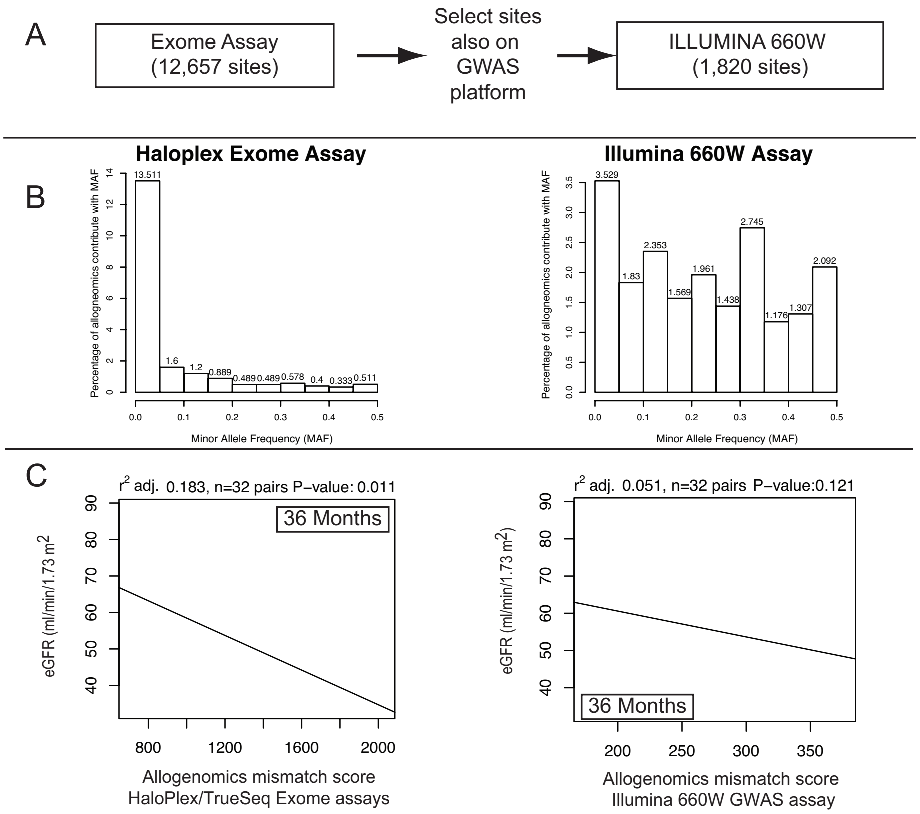

Figure D. Effect of genotyping platform on future replication studies. In this analysis, we estimate how well the allogenomics mismatch score could be evaluated with the genotyping array technology frequently used in GWAS studies. Analyses are done on the combined Discovery and Validation cohorts (n=32 pairs with 36-month eGFR, 64 exomes). Panel **A**) The allogenomics mismatch score evaluated with the Illumina TrueSeq or Agilent Haloplex exome platforms takes advantage of 12,657 genomic sites to estimate allogenomic contributions in transmembrane proteins. Only sites where an allogenomics mismatch score contribution different from zero are counted. We filtered the exome genomic sites to exclude sites not found on the Illumina 660W genotyping platform (used in [36]). After filtering, the allogenomics score is estimated with 1,820 remaining genomic sites. Panel **B**) The minor allele frequency (MAF) of the alleles described at each set of genomic sites is shown as a histogram (MAF is estimated from the Exac database, see Methods). Exome sequencing is an assay that directly observes variations in an individual DNA sample. The MAF distributions confirm that exome sequencing helps estimate contributions from many rare (MAF<5%) polymorphisms, whereas the chip genotyping platform estimates the score based on contributions from frequent alleles. Panel **C**) The scatterplot of the relationship between 36-month eGFR and the score estimated from the exome sites, or the subset of sites also measured by the GWAS platform. While some trend is still visible with sites measured on the GWAS platform, more samples would be needed to reach significance in the combined Discovery and Validation cohorts (n=34 pairs). Note that the magnitude of the scores is smaller on the GWAS platform because fewer contributions are summed. In contrast, the exome assays (Illumina TrueSeq for the Discovery cohort or Agilent Haloplex for the Validation cohort) result in stronger and significant correlations in the same set of samples.
